# HydroMoth as a tool for quantitative underwater SPL measurements in benthic ecological research

**DOI:** 10.1371/journal.pone.0352463

**Published:** 2026-06-24

**Authors:** Til Böttner, René Ortmann, Farhan Pasolong, Wolfgang H. Kirchner, Stefan Herlitze, Mareike Huhn

**Affiliations:** 1 General Zoology and Neurobiology, Ruhr-University Bochum, Bochum, Germany; 2 Luminocean, Banda Islands, Indonesia; 3 Behavioural Biology and Biology Education, Ruhr-University Bochum, Bochum, Germany; King Abdulaziz University, SAUDI ARABIA

## Abstract

Quantitative passive acoustic monitoring in benthic settings requires conversion of recorded amplitudes into absolute sound pressure levels (SPL, dB re 1 µPa). We tested whether HydroMoth (HM) recorders can provide sufficiently accurate SPL estimates in the low-frequency band relevant to benthic marine invertebrates and ship noise. Nine units were calibrated by a single-point offset using a 1000 Hz reference tone in an anechoic chamber and subsequently compared to a factory-calibrated SoundTrap ST600 in a controlled playback experiment (100–1000 Hz). In the pool dataset (HM1–HM5), deviations ranged from −2.6 to +1.8 dB with mean absolute errors typically within ~1–3 dB across frequencies; field measurements (HM6–HM9) showed deviations of similar magnitude, with mean deviations ranging from −0.5 to +1.0 dB across frequencies and mean absolute errors between 0.9 and 2.4 dB. Device orientation was fixed and identical across setups, reflecting benthic use cases where orientation is constrained and orientation related sensitivity differences act as a constant factor within deployments. These results show that HydroMoth recorders, under defined benthic conditions and within 100–1000 Hz, can provide SPL estimates with deviations comparable to those observed between commercial reference systems. This enables quantitative use of low-cost recorders in applications where absolute accuracy within a few decibels is sufficient.

## Introduction

Passive acoustic monitoring (PAM) has become an essential tool in marine ecology, providing non-invasive access to biological, geophysical, and anthropogenic sound sources [1,2]. Acoustic signals convey key information about ecological processes, species interactions, and environmental conditions, and they allow researchers to assess temporal and spatial dynamics in marine ecosystems. Of particular importance is the low- to mid-frequency band between 100 and 1000 Hz, where many ecologically relevant signals are concentrated. This range includes fish vocalizations, sound production by invertebrates, and the dominant low-frequency components of shipping noise, all of which shape marine soundscapes and influence organismal behavior [3–6]. Reliable quantification of such sounds is, therefore, fundamental for understanding habitat quality, assessing anthropogenic impacts, and advancing conservation-oriented acoustic research.

For quantitative use, digital amplitude values recorded by autonomous devices (dBFS) must be converted into absolute sound pressure levels (SPL, dB re 1 µPa). This requires calibration of the entire recording chain to enable meaningful comparisons across devices, studies, and ecological contexts. Controlled anechoic environments provide a practical means of obtaining such reference measurements while minimizing reflection artefacts. Standard recorders such as the SoundTrap ST600 achieve this through factory end-to-end calibration, but many widely used low-cost alternatives lack such traceable references. The HydroMoth (Open Acoustic Devices, UK) has nevertheless proven useful for detecting ecologically relevant signals, including reef soundscapes and fish sounds [1]. Its suitability for quantitative SPL analysis, however, depends on the availability of simple and reproducible calibration procedures.

Several calibration approaches have been described. Two-point calibrations using reference tones at different levels can yield accurate transfer functions under controlled conditions [7], whereas single-point offset calibrations offer a simplified alternative that requires only one reference measurement [8]. Portable, sound-dampened setups have also been suggested as a practical option for low-cost applications [9]. Among these, single-point methods are particularly attractive in ecological practice, as they minimize technical demands while still providing accuracy sufficient for many applications.

In this study, we investigate whether single-point calibration in an anechoic environment is sufficient for HydroMoth devices to provide reliable quantitative SPL measurements in the benthos range. Nine HydroMoth units were individually adjusted with a 1000 Hz reference tone in an anechoic chamber and subsequently tested in a controlled playback experiment against a factory-calibrated SoundTrap ST600. Five devices were tested in the pool and four devices in the sea. Our aim was to determine whether this straightforward and low-effort approach produces measurement accuracy adequate for ecological studies, thereby facilitating broader use of HydroMoth recorders in quantitative marine bioacoustics.

## Materials and methods

### Hydrophone systems

Nine HydroMoth recorders were used for the study, mounted in waterproof housings for underwater use. Each device integrates a MEMS-based hydrophone with a fixed preamplifier and a digital recording unit that stores signals in WAV format on an SD card. All experiments were conducted in low-gain mode to ensure high dynamics and a inexpensive signal-to-noise ratio at moderate sound pressure levels. The sampling rate was set to 48 kHz.

There is no factory-specified frequency response for the HydroMoth. However, previous evaluations in laboratory and field environments indicate that transmission in the low-frequency range (< 2 kHz) is comparatively stable, while above this spectrum, frequency-dependent deviations may increasingly occur [1].

Since HydroMoth devices do not feature factory end-to-end calibration and differences in sensitivity between individual units may arise due to manufacturing tolerances, an individual offset determination was performed for each device. This procedure makes it possible to convert the recorded relative levels into absolute sound pressure levels (dB re 1 µPa) and thus make direct comparisons with a calibrated reference system. In this study, individual offset correction was used not only to standardise HydroMoth outputs, but also to quantify device-specific deviations, which are of central importance for use in long-term ecological studies.

To have a comparison to a factory-calibrated device, a SoundTrap ST600 [10], was tested next to the HydroMoths. This system features end-to-end calibration, which includes the hydrophone, preamplifier and A/D converter, and provides absolute sound pressure levels (SPL, dB re 1 µPa) without additional correction steps. The manufacturer specifies a sensitivity of −189.2 dB re 1 V µPa ⁻ ¹ in low-gain mode for the device used. The frequency response is largely linear (±2 dB) in the range from 20 Hz to 20 kHz, so the ST600 was used in this experiment as a robust reference for validating the HydroMoth recordings.

### Calibration in an anechoic chamber

Calibration of the HydroMoths was performed in an anechoic chamber at Ruhr University Bochum, which minimises reflections thanks to wide absorber walls, thus creating a defined acoustic environment. A Brüel & Kjær 4230 sound level calibrator was used as the calibration source. This precision device generates a stable 1000 Hz sine tone at 94 dB SPL re 20 µPa (in air). This value corresponds to a reference level of 120 dB re 1 µPa in water. The HydroMoth devices were connected to the calibrator one after the other using a custom-fit adapter to ensure an airtight seal. This ensured that no acoustic leaks or positional shifts influenced the result. Several repetitions with 10 seconds of stable sound recording were performed for each device.

The raw data was evaluated using Audacity (version 3.7.4, The Audacity Team). The root mean square (RMS) amplitude of the signal in dBFS was calculated from each recording. An individual offset value was determined for each HydroMoth, representing the difference between the known reference level of the calibrator and the measured dBFS. This offset was then applied to all measurements from the same device so that their data could be converted to absolute sound pressure levels (dB re 1 µPa).

### Device orientation and application context

HydroMoth units are not omnidirectional, and device orientation can affect recorded amplitude. This limitation is known and relevant for free-field acoustic surveys [11]. However, the present study targets benthic applications, where marine invertebrates reside at or near the substrate and recording units are typically deployed in a fixed position relative to the seafloor and the sound field. Under such conditions, fixed device orientation reduces one important source of variability by maintaining a consistent geometric relationship between the recorder and the sound source. However, this does not replace a full characterisation of device directionality, and natural underwater sound fields are typically complex and multidirectional. The results presented here, therefore, apply primarily to stable and well-defined deployment geometries and should not be generalised to more heterogeneous acoustic environments. All HydroMoths in both the laboratory and the field experiments were mounted in the same horizontal orientation towards the acoustic axis of the source. The calibration offsets and subsequent deviations therefore reflect the accuracy achievable under realistic benthic deployment geometries, rather than general open water use cases.

### Playback experiment

**Fig 1:**
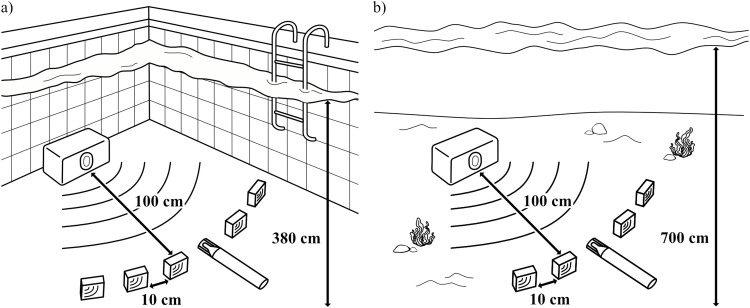
Schematic diagram of the underwater acoustic calibration test. **A)** A waterproofed BLIX Forever loudspeaker (model BS-850) emitted pure tone signals at 100, 200, 500 and 1000 Hz in a controlled swimming pool environment. Five HydroMoth units (HM1–HM5) and a SoundTrap ST600 reference hydrophone were placed 100 cm from the sound source in a linear array, all aligned along the acoustic axis with a distance of 10 cm to each other. This configuration ensured uniform exposure to the sound field and allowed a direct comparison of the recording performance of all devices. **B)** Equivalent field setup in a shallow marine environment, using the same source–receiver geometry and device spacing. Hydrophones were deployed at the seafloor, and distances to lateral and bottom boundaries exceeded 10 m to reduce reflections from solid structures. From left to right: HM5, HM4, HM3, SoundTrap ST600, HM2, HM1, HM6, HM7, SoundTrap ST600, HM8, HM9.

To verify the calibration, controlled signals were played back via an underwater loudspeaker (BLIX Forever BS-850), a commercially available portable Bluetooth speaker (nominal output power 10 W) not designed for underwater acoustic measurements and without a specified underwater frequency response. For underwater use, the loudspeaker was sealed with a standard food-grade vacuum sealing film (multi-layer PA/PE, transparent, nominal thickness 170/90 µm ± 10%), selected for its puncture resistance and barrier properties. Because the loudspeaker was not waterproof in its original configuration, all underwater playback trials were conducted with the sealed setup. A direct underwater comparison between sealed and unsealed configurations was therefore not feasible. Instead, playback fidelity was assessed indirectly by spectral verification of the reproduced signals (see section “Playback signal verification”). The experiment was conducted in the indoor swimming pool of the “Hallenbad Querenburg” (WasserWelten Bochum), which features a 50 m × 25 m competition pool with a depth range of 1.30–3.80 m. All measurements were taken at the deepest section of the basin, centrally along the 25 m width, to ensure consistent hydrostatic conditions and to minimise boundary reflections. Pure sine tones at 100, 200, 500 and 1000 Hz, generated with constant amplitude and a duration of 20 seconds, were used as stimuli. The signals were synthesised in Audacity, saved as uncompressed WAV files at a sampling rate of 44.1 kHz, and stored on an SD card inserted into the loudspeaker, which played the sound files sequentially during the experiment. Playback was performed locally via the SD card. No wireless transmission (e.g., Bluetooth) was used and no lossy compression applied during the experiments. A stable 5-second excerpt from the middle of the signal was evaluated from each recording to exclude transient and decay effects. The acoustic test setup consisted of the loudspeaker and the hydrophones, which were arranged in a line on the sound axis at 1 m distance from the loudspeaker. Five HydroMoth devices (HM1–HM5) and the SoundTrap ST600 as a reference were installed at the same depth and with identical orientation. The distance between the sensors was 10 cm (Fig 1A). Several repetitions were performed for each frequency per device: four measurements at 100 Hz and five each at 200, 500 and 1000 Hz. The water temperature remained constant at 26 ± 0.5 °C during all measurements.

### Field placement

A repeat calibration test was conducted in a marine environment at a depth of 7 m on sandy slope (Central Moluccas, Indonesia; 4.52305° S, 129.89783° E) using the same playback geometry and four additional HydroMoth (HM6–HM9) units together with the SoundTrap ST600. The loudspeakers and recording devices were positioned in open coastal waters at a distance of at least 10 m from nearby lateral and bottom boundaries, thereby reducing the influence of reflections from solid structures. As in the pool experiment, reflections from the water surface and associated multipath effects could not be avoided. The field setup therefore did not eliminate acoustic boundary effects, but reduced confinement-related reflections from nearby lateral and bottom structures compared with the pool environment. The chosen setup was intended to minimise, but not eliminate, boundary-related acoustic interference (Fig 1B). As in the controlled pool experiment, orientation of the devices was kept constant. The playback stimuli, offset determination and analysis were identical to those in the laboratory experiment. The field setup thus replicated the source – receiver geometry and device configuration used in the controlled pool experiment. Field recordings were conducted by an Indonesian researcher (co-author of the study) who did not need a permit for the implementation at the site. The test site was neither located inside a marine park nor in any other restricted zone requiring a permit.

### Playback signal verification

To assess the spectral integrity of the sealed playback configuration, we conducted an additional frequency-domain verification of the playback chain used in the validation experiment. Because the loudspeaker was not waterproof in its original configuration, it was covered with a thin plastic sealant for all underwater playback trials. A direct underwater comparison between sealed and unsealed conditions was therefore not feasible. For verification, replicate recordings of the playback tones were obtained with the SoundTrap ST600 under the same geometric setup used in the validation experiment. Four SoundTrap recordings were analysed for each target frequency (100, 200, 500, and 1000 Hz). In addition, one exemplary HydroMoth recording was analysed for each target frequency to confirm that the same overall spectral structure was present in the low-cost recorder data. All WAV files were processed in R using tuneR [12], seewave [13] and base FFT routines. If needed, stereo recordings were converted to mono. Prior to spectral analysis, a Hanning window was applied to each signal to reduce spectral leakage. One-sided amplitude spectra were then calculated and expressed in relative dB, normalised to the maximum amplitude within each recording. For each file, the dominant spectral peak was identified within a frequency window of ±20 Hz around the nominal target frequency. In addition, the strongest side peak between 0 and 2000 Hz was determined outside a guard band of ±15 Hz around the main peak. From these measurements, the detected peak frequency, the peak error relative to the nominal playback frequency, the frequency of the strongest side peak, and the difference in level between the main peak and the strongest side peak were calculated.

### Data processing and statistics

The raw data from the HydroMoth recorders was first extracted as digital amplitude values in decibels relative to full scale (dBFS). This was then converted into absolute sound pressure levels (SPL, dB re 1 µPa) based on the single-point offsets determined in the anechoic chamber. This calibration allowed all recordings to be converted into a physically comprehensible unit and compared directly with the measurements of the SoundTrap ST600. The difference between HydroMoth and the reference was determined for each individual measurement. Because pool and field measurements were conducted in separate sessions, SoundTrap reference SPL values were computed separately for each environment by averaging SoundTrap SPL across repetitions at each frequency (SoundTrapPool and SoundTrapSea). HydroMoth deviations were then calculated relative to the corresponding environment-specific reference (HydroMoth – SoundTrap). These deviation values formed the basis for all further analyses and allowed both an assessment of systematic tendencies (overestimation or underestimation of the sound pressure level) and a characterisation of the variability between devices and frequencies.

The statistical analysis was performed in two steps. First, descriptive parameters were calculated, including mean, standard deviation, interquartile range (IQR) and mean absolute error (MAE). These measures quantify the central tendency and the dispersion of the deviations and allow an initial assessment of the measurement accuracy of the HydroMoth devices. To systematically investigate the influencing factors, a linear mixed-effects model was calculated. In this model, the SPL deviation was modelled as a dependent variable, the test frequency was considered as a fixed effect and the device identity as a random effect. In this way, repeated measurements of the same units could be statistically correctly mapped and frequency-specific differences in the deviation quantified. The significance tests of the model parameters were based on the Satterthwaite approximation of degrees of freedom. Post-hoc analyses with Tukey correction were used to test frequency-specific differences in pairs. Model quality was assessed using marginal and conditional R² to capture both the variance explained by fixed effects and the contribution of interindividual differences between HydroMoths. In addition, effect sizes were calculated, including partial Eta² for the main effects and standardised mean differences (Cohen's d with Hedges’ correction) for the contrasts. All statistical analyses were conducted in R [14]. Data preparation and statistical modelling were performed using the packages dplyr [15], lme4 [16] and emmeans [17]. Model performance assessment and the calculation of standardized effect sizes were carried out using performance and effectsize [18]. Graphical representations were created using ggplot2 [19], patchwork [20]. Acoustic file processing and spectral analyses were conducted using tuneR [12] and seewave [13]. Additional data import, reshaping, and workflow functions were implemented using readxl [21], tidyr [22], tibble [23], stringr [24], and purrr [25].

## Results

We quantified deviations in sound pressure level (SPL) between HydroMoth recorders and a factory-calibrated SoundTrap ST600 across four test frequencies (100, 200, 500, and 1000 Hz) following single-point offset calibration. Measurements were conducted under two deployment conditions: a controlled pool experiment using five HydroMoth units (HM1–HM5; laboratory dataset, N = 95) and a field experiment in a shallow coastal marine environment with sandy bottom, using four additional units (HM6–HM9; field dataset, N = 80). The response variable throughout was the SPL deviation expressed as the difference between HydroMoth and SoundTrap measurements (HydroMoth – SoundTrap, in dB re 1 µPa). In the laboratory pool experiment, HydroMoth devices showed small deviations from the reference system across all frequencies (Table 1). Median deviations ranged from −0.41 dB at 100 Hz to −1.02 dB at 1000 Hz. The interquartile range (IQR) increased with frequency, from 2.28 dB at 100 Hz to 5.05 dB at 1000 Hz. Mean deviations were close to zero at 100 Hz (−0.29 dB) and 500 Hz (+0.12 dB), while larger negative mean deviations were observed at 200 Hz (−1.05 dB) and 1000 Hz (−1.53 dB). Mean absolute error (MAE) values ranged from 1.40 dB at 100 Hz to 2.75 dB at 200 Hz.

**Table 1:** Deviation of HydroMoth SPL measurements from the SoundTrap ST600 (laboratory test) Deviation of sound pressure level (SPL) of HydroMoth devices compared to the reference system (SoundTrap ST600), given in dB re 1 µPa. Median, interquartile range (IQR), mean deviation, standard deviation (SD), and mean absolute error (MAE) are reported for each test frequency.

Frequency (Hz)	n	Median (dB)	IQR (dB)	Mean (dB)	SD (dB)	MAE (dB)
100	20	−0.41	2.28	−0.29	1.71	1.40
200	25	−1.88	4.26	−1.05	2.97	2.75
500	25	−0.23	2.42	0.12	1.77	1.42
1000	25	−1.02	5.05	−1.53	3.29	2.74

In the field experiment, deviations were of comparable magnitude but exhibited slightly different central tendencies (Table 2). Median deviations ranged from −0.35 dB at 200 Hz to +0.85 dB at 500 Hz. Mean deviations were positive at 100 Hz (+0.33 dB), 200 Hz (+0.17 dB), and 500 Hz (+1.00 dB), and slightly negative at 1000 Hz (−0.45 dB). Dispersion was lowest at 100 Hz (IQR = 0.80 dB, SD = 1.17 dB) and highest at 1000 Hz (IQR = 4.38 dB, SD = 3.24 dB). Mean absolute error ranged from 0.87 dB at 100 Hz to 2.38 dB at 1000 Hz.

**Table 2:** Deviation of HydroMoth SPL measurements from the SoundTrap ST600 (field test in marine environment) In situ measurements conducted under field conditions in a coastal marine environment. Values represent deviations in dB re 1 µPa between HydroMoth devices and the calibrated reference hydrophone ST600.

Frequency (Hz)	n	Median (dB)	IQR (dB)	Mean (dB)	SD (dB)	MAE (dB)
100	20	+0.35	0.80	+0.33	1.17	0.87
200	20	−0.35	4.21	+0.17	2.97	2.13
500	20	+0.85	1.87	+1.00	1.56	1.17
1000	20	+0.06	4.38	−0.45	3.24	2.38

**Fig 2:**
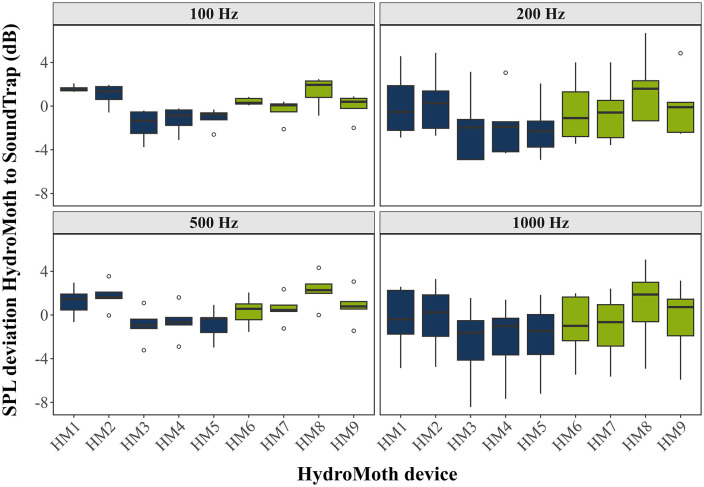
Deviation in sound pressure level (SPL) between HydroMoth and SoundTrap measurements across test frequencies (100–1000 Hz). Boxplots show SPL differences (HydroMoth – SoundTrap) in dB re 1 µPa for nine HydroMoth devices (HM1–HM9) after single-point offset calibration in an anechoic chamber. Devices HM1–HM5 were tested in a controlled pool environment (laboratory test, N = 95), whereas HM6–HM9 were deployed under field conditions in a shallow seaside habitat (field test, N = 80). Blue boxplots indicate pool (laboratory) measurements, whereas green boxplots indicate sea (field) measurements. Positive deviation indicate overestimation, negative deviations underestimation relative to the SoundTrap ST600. Sample sizes per frequency and environment are provided in Table 1 (laboratory) and Table 2 (marine field test).

Across both environments, SPL deviations were generally centered within a few decibels of the reference, with no single frequency showing disproportionately large errors (Fig 2).

**Fig 3:**
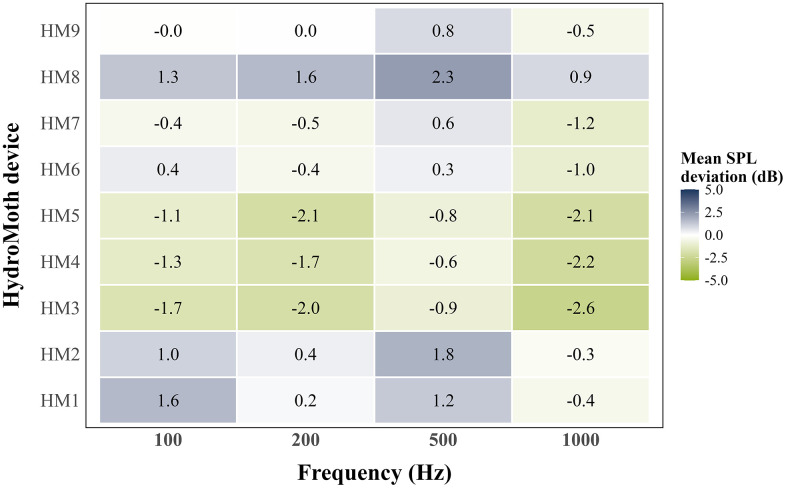
Mean sound pressure level (SPL) deviation of HydroMoth devices relative to the SoundTrap ST600 across test frequencies. The heatmap shows the mean difference (HydroMoth – SoundTrap, in dB re 1 µPa) for each HydroMoth device (HM1–HM9) and frequency (100, 200, 500, 1000 Hz) after single-point offset calibration in an anechoic chamber. Green shades indicate underestimation and blue shades indicate overestimation of SPL relative to the reference. Numerical values in each cell represent the mean deviation rounded to one decimal place. Devices HM1–HM5 were tested in a controlled pool environment (laboratory test; N = 95), while HM6–HM9 were measured under field conditions in a shallow coastal marine environment (field test; N = 80).

Mean deviations calculated for each HydroMoth device and frequency revealed a high degree of consistency among devices (Fig 3). Across most device-frequency combinations, mean deviations clustered close to zero. Slight positive offsets at 500 Hz and slight underestimations at 1000 Hz were observed in both environments, whereas deviations at 200 Hz showed greater spread among devices without being dominated by individual outliers. Devices tested in the pool (HM1–HM5) and those tested in the field (HM6–HM9) exhibited overlapping ranges of mean deviations, indicating comparable performance under laboratory and field conditions. The comparatively higher variability observed at 200 Hz did not follow a consistent pattern across devices or environments and was not driven by individual outliers. As this pattern was not consistently observed across all frequencies, the data do not indicate a systematic bias specific to 200 Hz. To formally assess the effects of frequency and deployment environment on SPL deviation while accounting for repeated measurements per device, we fitted a linear mixed-effects model with frequency, environment (pool vs sea), and their interaction as fixed effects, and device identity as a random intercept: spl_diff ~ Frequency × environment + (1 | device).

The model revealed a significant main effect of frequency on SPL deviation (Type III ANOVA: F(3, 159.97) = 3.51, p = 0.0168). In contrast, the main effect of environment was not significant (F(1, 6.97) = 1.69, p = 0.235), and there was no evidence for a frequency-by-environment interaction (F(3, 159.97) = 0.13, p = 0.942).

Variance components indicated that between-device differences accounted for a relatively small proportion of the total variance (random intercept variance = 0.91 dB², SD = 0.95 dB), while most variability was attributable to residual variation within devices (residual variance = 5.50 dB², SD = 2.35 dB). Model fit statistics reflected this pattern, with a marginal R² of 0.084 and a conditional R² of 0.214.

Estimated marginal means and Tukey-adjusted pairwise comparisons showed no statistically significant differences between individual frequency levels within either environment. Comparisons between pool and field conditions at each frequency likewise revealed no significant differences (all p > 0.21). Effect size estimates supported these findings: partial eta squared for the frequency effect was 0.06, indicating a small effect, while the effect size for environment and the interaction term was negligible.

Overall, these analyses demonstrate that HydroMoth SPL deviations relative to the SoundTrap reference are small and consistent across devices, with modest frequency-dependent shifts and no systematic difference between controlled laboratory and shallow marine field conditions.

### Spectral verification of the sealed playback configuration

**Fig 4:**
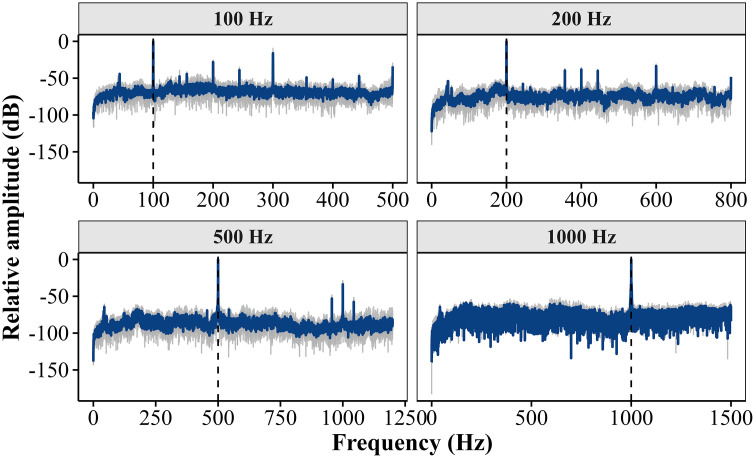
Spectral fidelity of the sealed playback configuration across the test frequencies. Mean power spectra (blue lines) and standard deviation (shaded areas) of repeated SoundTrap ST600 recordings are shown for playback tones at 100, 200, 500, and 1000 Hz. Individual replicates are plotted as thin grey lines. Dotted vertical lines indicate the nominal playback frequencies. Across all frequencies, dominant spectral peaks occurred at the intended target frequencies with minimal deviation. At 100 Hz, an additional spectral component was observed at approximately 300 Hz, resulting in reduced peak-to-sideband separation compared with the higher frequencies. Nevertheless, the target frequency remained clearly identifiable in all cases, indicating that the sealed playback configuration did not introduce major spectral distortion within the tested range.

Spectral verification of the sealed playback configuration showed that the intended playback frequencies were reproduced with high precision across SoundTrap replicates. The dominant spectral peak was detected at the nominal target frequency in all cases (Fig 4). Mean peak errors were very small, amounting to 0.0106 Hz at 100 Hz, 0.0212 Hz at 200 Hz, −0.0448 Hz at 500 Hz, and 0.0173 Hz at 1000 Hz. Spectral separation between the dominant peak and the strongest side peak was high for 200–1000 Hz, with mean differences of 33.5 dB at 200 Hz, 31.6 dB at 500 Hz, and 47.6 dB at 1000 Hz. At 100 Hz, the mean peak-to-side peak separation was lower (16.1 dB), and the strongest side peak occurred consistently at 300 Hz in three of four SoundTrap replicates. An analogous side peak at 300 Hz was also observed in the exemplary 100 Hz HydroMoth recording. Despite this reduced spectral separation at the lowest test frequency, the dominant peak remained clearly centered at 100 Hz in all recordings.

## Discussion

The results of this study demonstrate that HydroMoth recorders, following a single-point offset calibration, can measure underwater sound pressure levels in the range of 100–1000 Hz with mean deviations of approximately ~1–3 dB relative to a factory-calibrated reference system (SoundTrap ST600). This level of accuracy is comparable to that reported for several commercially available passive acoustic monitoring systems [26]. Across the tested frequency spectrum, deviations were generally consistent. Slight overestimation was observed around 500 Hz, whereas a tendency towards underestimation occurred at 1000 Hz. Variability was also somewhat higher at 200 Hz, although this pattern was inconsistent across devices and environments. These small frequency-dependent deviations may arise from multiple, non-exclusive factors. First, the frequency response of the HydroMoth sensors is not fully characterised and may exhibit subtle deviations across the tested range. Second, the single-point offset calibration approach assumes a constant sensitivity offset across frequencies, which may not fully capture frequency-dependent variation in the recording chain. Third, the playback system itself may contribute to minor spectral imbalances due to its transfer characteristics, particularly under the modified (sealed) configuration required for underwater use. Finally, residual effects of the experimental geometry, including reflections and interference patterns, may influence the recorded amplitudes in a frequency-specific manner. In autonomous marine recorders, such effects may also arise from interactions between the incoming sound field and the recorder body itself, which can produce frequency- and angle-dependent sensitivity differences [27]. As no consistent monotonic trend across frequencies was observed, the present data do not support a single dominant mechanism but rather suggest that the observed deviations likely result from a combination of these factors. The magnitude of these deviations, however, remains small relative to the overall measurement accuracy.

The linear mixed-effects model revealed a small but statistically significant main effect of frequency on SPL deviation, while neither the deployment environment (pool versus sea) nor the frequency-by-environment interaction showed a significant effect. The random intercept for device identity accounted for only a minor proportion of the total variance, indicating high agreement among individual HydroMoth units. Most of the variation was attributable to repeated measurements within devices rather than systematic differences between devices.

Deviations observed under field conditions were comparable in magnitude to those measured in the controlled pool experiment, and the statistical model provided no evidence for a systematic environment effect. This indicates that the accuracy achieved under laboratory conditions is transferable to shallow marine field deployments when geometry and playback conditions are matched. The applicability of the present findings is therefore limited to low-frequency SPL measurements obtained under defined and stable source-receiver configurations. Deployments involving variable orientation, complex sound fields, or higher-frequency signals require further validation.

The present study does not assess HydroMoths as general-purpose hydrophones. Device directionality is known to influence recorded amplitude, and this characteristic constrains the scope of the conclusions. Previous work has shown that hydrophone orientation may affect received sound levels in field settings, although the magnitude of this effect depends on the acoustic environment and deployment configuration [11]. The results presented here apply specifically to benthic applications, where recorders are typically deployed in a fixed orientation relative to the substrate and the sound field. Under such conditions, fixed device orientation reduces one important source of variability by maintaining a consistent geometric relationship between the recorder and the sound source. However, hydrophones deployed close to the seafloor may exhibit angle- and frequency-dependent receive characteristics influenced by substrate interactions and boundary effects, which can modify the effective sensitivity depending on the arrival direction of the sound field [28]. Nevertheless, natural underwater sound fields are typically complex and multidirectional, and device directionality may still influence recorded amplitudes under less controlled conditions. In addition, recent evaluations of compact autonomous underwater recorders have shown that their effective directivity can vary substantially with frequency and angle of incidence due to interactions between the incoming sound field and the recorder housing, leading to non-negligible deviations from ideal omnidirectional behaviour [29]. The results presented here therefore apply primarily to defined and stable deployment geometries and should not be interpreted as a full characterisation of HydroMoth performance in heterogeneous acoustic environments. The tested frequency range of 100–1000 Hz reflects a spectral domain relevant to benthic invertebrate bioacoustics, as marine invertebrates primarily detect particle motion at frequencies below 1000 Hz and overlaps with the dominant low-frequency components of ship noise. The range does not imply general suitability beyond these frequencies [30,31]. The SoundTrap ST600, although factory-calibrated, also carries its own measurement uncertainty and does not constitute a metrological reference standard. Likewise, the single-point offset calibration employed here is not intended as an alternative to IEC-compliant hydrophone calibration.

Formal hydrophone calibration is governed by international standards such as IEC 60565–1:2020 and IEC 60565–2:2019 [32], which specify laboratory-based procedures using traceable reference hydrophones and controlled acoustic environments. The present study does not aim to meet these standards. Instead, it proposes a pragmatic approximation approach tailored to ecological applications in which full metrological calibration is impractical, but high replication and spatial coverage are essential.

From an ecological perspective, the primary significance of these findings lies in enabling quantitative SPL-based analyses using low-cost recording devices. The frequency range examined here encompasses many biologically and ecologically relevant signals, including sounds produced by benthic invertebrates, fish vocalisations, and the low-frequency components of shipping noise [3,6,33]. Such signals are widely used as indicators of anthropogenic disturbance and ecological condition [5,34,35]. The close correspondence between HydroMoth and SoundTrap measurements suggests that HydroMoths can be used in ecological monitoring programmes that require quantitative estimates of sound pressure levels. Their low cost allows deployment at spatial scales that would be prohibitive with high-end reference systems. Previous studies have demonstrated the suitability of HydroMoths for detecting biological and anthropogenic sounds [1]; the present study extends this work by showing that their recordings can be standardized to absolute SPL units with relatively small uncertainty.

Several limitations remain. The calibration procedure relied on a single reference tone obtained under controlled laboratory conditions. The long-term stability of the derived offsets under varying environmental conditions, including changes in temperature, pressure, and ambient noise, remains to be tested.

In addition, the playback system required waterproofing of the loudspeaker using a thin plastic sealant. Because an unsealed underwater configuration was not feasible, spectral verification of the playback signals was performed. This analysis confirmed stable reproduction of the intended frequencies across the tested range. Although reduced spectral separation was observed at 100 Hz, indicating minor low-frequency artefacts, there was no evidence for substantial distortion of the playback signals. The calibration results presented here are therefore unlikely to be driven by artefacts of the playback system.

Furthermore, the present analysis was restricted to frequencies up to 1000 Hz, and further validation is required for higher-frequency applications, such as odontocete vocalizations or active sonar signals.

At the lowest test frequency of 100 Hz, the acoustic wavelength in water is approximately 15 m, placing the 1 m source-receiver distance well within the near field. Near-field conditions can theoretically produce complex pressure gradients and non-uniform sound propagation. However, the present data do not show increased deviation magnitudes or variance at 100 Hz compared to higher frequencies. Both mean deviations and dispersion remained within the same range observed across the spectrum. This suggests that any near-field effects under the specific test geometry were either negligible or consistent across devices and therefore did not compromise comparability. Replication in open coastal waters, where devices were positioned more than 10 m from reflective boundaries and thus under more defined far-field conditions, yielded deviation magnitudes of similar size, further supporting the robustness of the results at low frequencies.

## Conclusion

This study demonstrates that HydroMoth recorders, when individually calibrated using a single-point offset in an anechoic chamber, can provide quantitative underwater sound pressure level measurements with deviations of approximately 2–3 dB relative to a factory-calibrated SoundTrap ST600. This level of agreement was consistent across the tested frequency range of 100–1000 Hz, which encompasses many acoustically and ecologically relevant signals in benthic marine environments. Importantly, deviations observed under controlled laboratory conditions in a swimming pool were comparable to those measured during a replicated field deployment in shallow coastal waters. The absence of a significant environment effect indicates that, under matched source-receiver geometry and fixed device orientation, calibration performance obtained in the laboratory can be transferred to realistic benthic field settings. Device-to-device variability was small, and most of the observed variation arose from repeated measurements within devices rather than systematic differences among units. The results presented here do not suggest that HydroMoths constitute a substitute for formally calibrated hydrophones in a metrological sense. Instead, they demonstrate that a pragmatic, low-effort calibration approach can yield absolute SPL estimates of sufficient accuracy for many ecological applications, particularly where relative differences, spatial patterns, or temporal trends in sound levels are of primary interest. The method is therefore well suited for studies that require high replication, spatial coverage, or long-term deployments but lack access to high-cost reference systems.
